# Association Study of Germline Variants in *CCNB1* and *CDK1* with Breast Cancer Susceptibility, Progression, and Survival among Chinese Han Women

**DOI:** 10.1371/journal.pone.0084489

**Published:** 2013-12-27

**Authors:** Yan Li, Yi-Lin Chen, Yun-Tao Xie, Li-Yuan Zheng, Ji-Yuan Han, Hui Wang, Xin-Xia Tian, Wei-Gang Fang

**Affiliations:** 1 Department of Pathology, Key Laboratory of Carcinogenesis and Translational Research (Ministry of Education), School of Basic Medical Sciences, Peking University Health Science Center, Beijing, China; 2 Breast Center, Peking University School of Oncology, Beijing Cancer Hospital & Institute, Beijing, China; MOE Key Laboratory of Environment and Health, School of Public Health, Tongji Medical College, Huazhong University of Science and Technology, China

## Abstract

The *CCNB1* and *CDK1* genes encode the proteins of CyclinB1 and *CDK1* respectively, which interact with each other and are involved in cell cycle regulation, centrosome duplication and chromosome segregation. This study aimed to investigate whether the genetic variants in these two genes may affect breast cancer (BC) susceptibility, progression, and survival in Chinese Han population using haplotype-based analysis. A total of ten tSNPs spanning from 2kb upstream to 2kb downstream of these genes were genotyped in 1204 cases and 1204 age-matched cancer-free controls. The haplotype blocks were determined according to our genotyping data and linkage disequilibrium (LD) status of these SNPs. For *CCNB1*, rs2069429 was significantly associated with increased BC susceptibility under recessive model (OR=2.352, 95%CI=1.480-3.737), so was the diplotype TAGT/TAGT (OR=1.947 95%CI=1.154-3.284, P=0.013). In addition, rs164390 was associated with Her2-negative BC. For *CDK1*, rs2448343 and rs1871446 were significantly associated with decreased BC risk under dominant models, so was the haplotype ATATT. These two SNPs also showed a dose-dependent effect on BC susceptibility. Using stratified association analysis, we found that women with the heterozygotes or minor allele homozygotes of rs2448343 had much less BC susceptibility among women with BMI<23. In *CDK1*, three closely located SNPs, rs2448343, rs3213048 and rs3213067, were significantly associated with tumor’s PR status: the heterozygotes of rs2448343 were associated with PR-positive tumors, while the minor allele homozygotes of rs3213048 and heterozygotes of rs3213067 were associated with PR-negative BC tumors. In survival analysis, rs1871446 was associated with unfavorable event-free survival under recessive model, so was the *CDK1* diplotype ATATG/ATATG, which carried the minor allele homozygote of rs1871446. Our study indicates that genetic polymorphisms of *CCNB1* and *CDK1* are related to BC susceptibility, progression, and survival in Chinese Han women. Further studies need to be performed in other populations as an independent replication to verify these results.

## Introduction

Breast cancer (BC) has become the most common cancer affecting women all around the world; its incidence also ranks first among female cancers in China [1]. Several breast cancer predisposition genes have been identified such as low-frequency, high-penetrance genes BRCA1, BRCA2, PTEN and p53, as well as low-frequency, intermediate-penetrance genes CHEK2, ATM and PALB2 [2]. However, these mutations explain only a small proportion of the total genetic risk of BC. As a common complex disease, BC is also interpreted by high-frequency, low-penetrance genetic variation according to the “common disease, common variants” hypothesis [3]. 

Single nucleotide polymorphisms (SNPs), which amount to approximately 15 million in human genome [4], denote sites where the genomes of different people vary by a single base. Based on linkage disequilibrium (LD) theory [5], a set of informative SNPs (tag SNPs) can capture the contribution of the whole SNPs in a region of a chromosome. Therefore, it is cost-effective to genotype tSNPs. A set of associated SNP alleles in a region of a chromosome is identified as a “haplotype”, while a pair of haplotypes forms a “diplotype”. Over the past ten years, several genome-wide association studies (GWASs) reported BC susceptibility variants at multiple loci in different populations [5-10].

CyclinB1 and *CDK1*, which are two crucial regulatory proteins of centrosome, can form a complex (M-phase promoting factor, MPF) and regulate the entry into mitosis [11], enhance chromosome condensation and nuclear envelope breakdown [12,13]. The overexpression of CyclinB1 has been found in brain astrocytoma, cervical carcinoma, lung cancer, and many other cancers [14,15]. *CCNB1* amplification was also reported in colorectal adenocarcinomas [16]. One study found that docetaxel could suppress the expression of *CCNB1* in non-small cell lung cancer NCI-H460 cells [17]. Hui Cai and colleagues found that rs2069433 in *CCNB1* was related to a reduction in endometrial cancer risk [18]. H Ma and colleagues reported that rs2069429 in *CCNB1* was associated with non-small cell lung cancer survival [19]. CDK1 overexpression has been found in human gliomas [20]. Previous research in our lab found that CyclinB1 and CDK1 were highly expressed in BC and associated with patients’ overall survival (unpublished data).

Based on the previous studies, we proposed the hypothesis that genetic variants in *CCNB1* and *CDK1* contributed to BC’s susceptibility, progression and patients’ survival. To examine this hypothesis, we selected 4 and 6 tSNPs to represent these two genes respectively, spanning from 2kb upstream to 2kb downstream of *CCNB1* and *CDK1* (chromosome 5:68,496,669...68,511,822 for *CCNB1*; chromosome 10:62,206,242…62,225,930 for *CDK1*). In this study, we comprehensively investigated the associations of tSNPs, haplotypes, and diplotypes in *CCNB1* and *CDK1* with BC susceptibility, clinicopathological parameters and event-free survival in Chinese Han population\.

## Materials and Methods

### Study Population

This study included 1204 female BC patients and 1204 cancer-free unrelated female individuals. All the 1204 patients were pathologically diagnosed with primary invasive ductal breast carcinoma from 1995 to 2007 in Beijing Cancer Hospital. Their epidemiological information was obtained from their clinical records, including age at diagnosis, height, weight, age at menarche and/or menopause, menopause status, age at first full-term pregnancy and family history of cancer in first-degree relatives. Body mass index (BMI) was calculated by weight and height, and was used to quantify obesity (BMI≥23 as overweight; BMI<23 as normal) [21]. All the clinicopathological parameters, including ER, PR, Her2, tumor size, lymph node status, and clinical stage (based on the 6th edition of TNM staging of the American Joint Committee on Cancer system), were also collected from their clinical records. The event-free survival time was defined as the time from the surgery to the breast events such as breast carcinoma recurrence, metastasis, and death caused by BC. Cases were censored if the patients were still alive or voluntarily withdrew or died of a cause other than BC before the latest follow-up (August 31, 2010). Of the 1204 cases, 48 cases had no surgery, 20 cases died of unknown causes, and 131 cases were lost to follow-up. Therefore, 1005 cases remained in the event-free survival analysis.

The 1204 cancer-free female individuals were selected from a community-based screening program for non-infectious diseases conducted in Beijing. The controls were age-matched to cases by 5-year age groups. All the epidemiological information was collected from the questionnaire they completed.

This study was approved by the Peking University IRB (reference No. IRB00001052-11029). Breast Cancer samples were collected initially for research purposes in the tissue/blood biobank. Written consents were collected from the BC patients who can read and write. Verbal consents were obtained from the BC patients who cannot read and write, however, for these cases, written consent was signed by her next of kin. Written consents were obtained from all control samples. The IRB approved the written consent procedure. The data/samples were used anonymously. PKU IRB approved our application to waive informed re-consent for the already collected BC samples in the tissue/blood biobank. This study only used this part of samples.

### SNPs selection

All SNPs in *CCNB1* and *CDK1* genes were selected according to the public HapMap database (HapMap Data Release 27; Chinese Beijing population) and the NCBI dbSNP database (dbSNP b126; Chinese Beijing population) (Website: http://hapmap.ncbi.nlm.nih.gov/) [22]. HapMap database is a public database and all the data they provided is anonymous. For *CCNB1*, a total of 14 common SNPs, with a minor allele frequency >0.05 , were selected, spanned from 2kb upstream to 2kb downstream of *CCNB1* gene (chromosome 5:68,496,669...68,511,822). For *CDK1*, 41 common SNPs were selected (chromosome 10:62,206,242...62,225,930). Using the Tagger algorithm [23] implemented in the HaploView software 4.2 [24], we identified 4 tSNPs in *CCNB1* (being rs350104, rs2069429, rs164390, rs2069433) and 6 tSNPs in *CDK1* (being rs2448343, rs3213048, rs3213067, rs1871446, rs10711, rs1060373) to best capture the common genetic variations within the genes.

### DNA isolation, genotyping assays, and quality control

Genomic DNA was isolated from blood leukocytes by proteinase K digestion followed by phenol-chloroform extraction and isopropanol precipitation. SNP genotyping was performed by Taqman Assay® (Appplied Biosystems, FosterCity, California) using the ABI Step One® Real-Time PCR System (Applied Biosystems, FosterCity, California). Primers and probes (FAM- and VIC- labeled) were directly supplied by Applied Biosystems as Assays-by-Design^TM^ or Assays-on-Demand^TM^ products. The PCR conditions were as same as that described by Yuan Ruan and colleagues [25]. Positive and negative controls were run together in every genotyping assay. At least 1% of samples were duplicated randomly in each SNP assay, and the concordance between duplicates was more than 99%.

### LD block determination and haplotype construction

The most probable haplotypes for each participant were estimated using the SAS9.1 PROC HAPLOTYPE procedure. According to the genotyping results of the tSNPs, Linkage Disequilibrium (LD) measured by Lewontin coefficient (D’) and squared correlation coefficient (r^2^) between the genotyped SNPs was calculated [24]. Then haplotype blocks in cases, controls, and all the participants were respectively reconstructed with the HaploView 4.2 software.

### Statistical analysis

For each tSNP, Hardy-Weinberg Equilibrium in control subjects was examined. Two-sided t test (for continuous variables) and chi-square (χ^2^) test (for categorical variables) were performed to determine the differences between cases and controls. Each tSNP was evaluated according to codominant, dominant, and recessive models [26]. Two-sided chi-square test was also used to investigate the differences in the distributions of genotypes between cases and controls, and to evaluate the association of alleles or genotypes with the clinical parameters. The effects of alleles or genotypes on breast carcinoma risk and progression were determined by odds ratios (OR) and 95% Confidence Intervals (95% CI) using both univariate and multivariate Logistical Regression models [25,27,28]. For gene-gene and gene-environment interaction analysis, we conducted stratified association analysis. Kaplan-Meier curves were generated for event-free survival, and log-rank statistics were also used to verify the survival curves. Both univariant and multivariant Cox’s proportional hazard model were used to determine the hazard ratio (HR) and the corresponding 95% CI. A two-sided P value<0.05 was considered statistically significant. All analyses were performed using Statistic Analysis System software (SAS v9.1, SAS Institute, Cary, NC).

## Results

### Characteristics of the population

The demographic data were analyzed by chi-square test (for categorical variables) and two-sided t test (for continuous variables) (Table S1). The cases and controls appeared to be adequately matched on age (P=0.437). As expected, the cases had a much younger age at menarche (P<0.0001), fewer number of births (P<0.0001) and an elder age at first full-term pregnancy (P<0.0001) than the controls. In addition, cases were more likely to have a family history of cancer (P=0.045) and a high BMI (P=0.015), and have been breastfeeding less than 6 months (P<0.0001). 

### LD degree between SNPs

**Figure 1:**
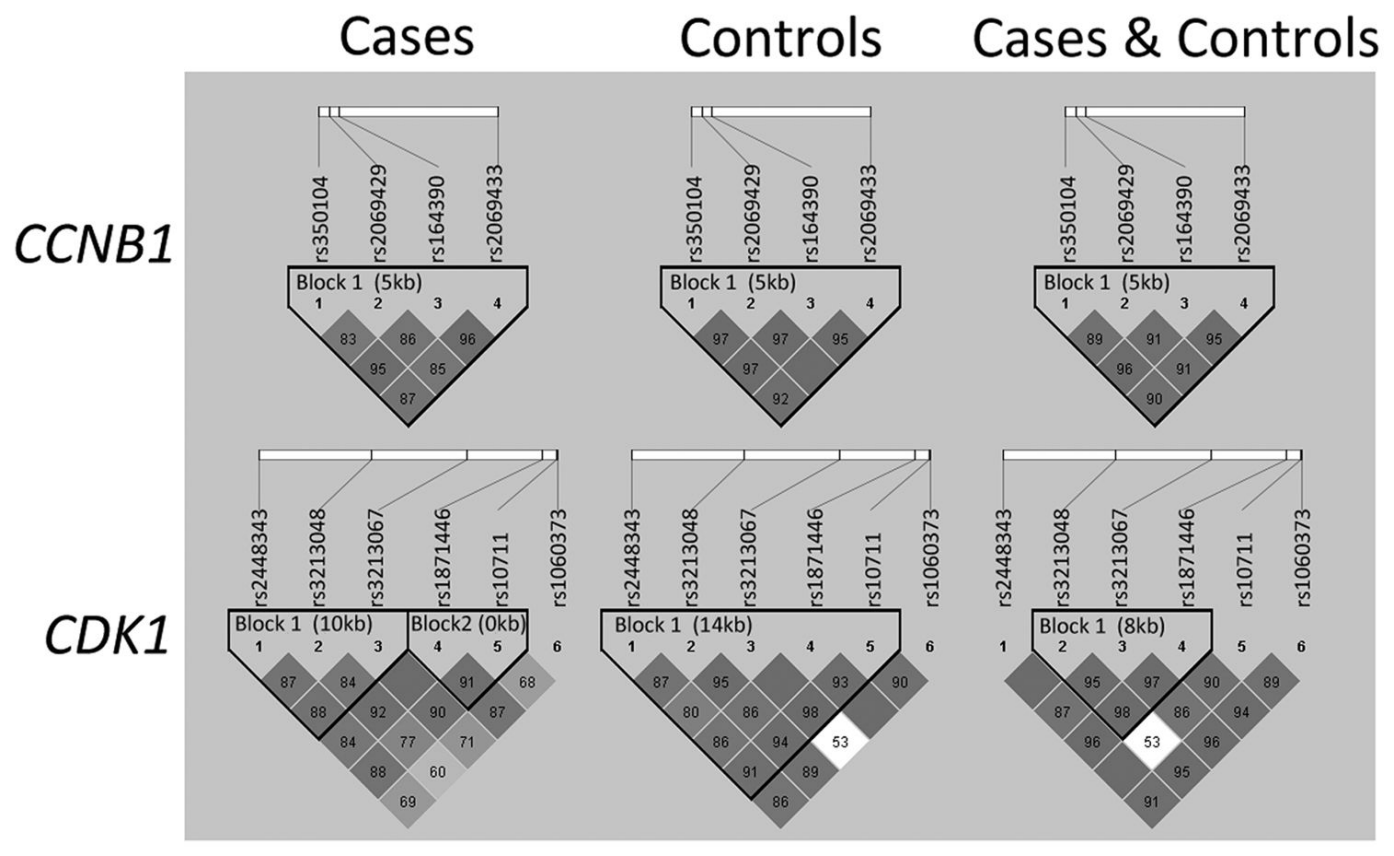
LD maps of the analyzed ten SNPs in controls and BC cases. The values shown in each diamond are the D’*100 (10 means 0.10, 1 means 0.01). Dark grey diamonds without a number indicate that the value of D’ is 1. The dark grey-to-white gradient reflects higher to lower LD values.

The 10 tSNPs were all in agreement with Hardy-Weinberg equilibrium (P>0.1) in the controls (Table S2). Table 1 illustrated the frequency distributions of alleles and genotypes for the ten tSNPs among cases and controls. The LD degree of all tSNPs in case population, control population, and total subjects (cases plus controls) were shown in Figure 1. The haplotype block of *CCNB1* in cases was consistent with that in control population, and thus the 4-SNP haplotype block was chosen (rs350104, rs2069429, rs164390, and rs2069433). However, in *CDK1*, the rs3213048 and rs 10711 were in strong LD in controls, but in weak LD in cases. Therefore, we chose the 5-SNP haplotype block for *CDK1* according to our genotyping data in controls (rs2448343, rs3213048, rs3213067, rs1871446, rs10711).

**Table 1:** Allele and genotype frequencies of the selected tSNPs in *CCNB1* and *CDK1* and the association with risk of breast cancer.

Gene	SNPs	Genotype	Cases (%)	Controls (%)	P*	P**	P_trend_	OR (95%CI)	aOR (95%CI)***
CCNB1	rs350104	TT	607 (50.42%)	586 (48.67%)	0.287		0.826		
		CT	481 (39.95%)	516 (42.86%)				0.900 (0.761-1.065)	0.900 (0.753-1.076)
		CC	116 (9.63%)	102 (8.47%)				1.098 (0.822-1.466)	0.976 (0.717-1.330)
		C allele frequency	0.296	0.299		0.825			
		(CC+CT) vs. TT (dominant model)						0.933 (0.795-1.094)	0.913 (0.771-1.082)
		CC vs. (CT+TT) (recessive model)						1.152 (0.871-1.522)	1.025 (0.761-1.380)
	rs2069429	GG	823 (68.36%)	857 (71.18%)	**0.001**		**0.011**		
		AG	320 (26.58%)	320 (26.58%)				1.041 (0.868-1.249)	1.035 (0.853-1.255)
		AA	61 (5.07%)	27 (2.24%)				**2.352 (1.480-3.737)**	**2.359 (1.449-3.840)**
		A allele frequency	0.184	0.155		**0.009**			
		(AA+AG) vs. GG (dominant model)						1.143 (0.961-1.361)	1.139 (0.947-1.370)
		AA vs. (AG+GG) (recessive model)						**2.326 (1.468-3.685)**	**2.338 (1.440-3.794)**
	rs164390	GG	346 (28.74%)	308 (25.58%)	0.176		0.285		
		TG	552 (45.85%)	590 (49.00%)				0.833 (0.687-1.010)	0.917 (0.748-1.124)
		TT	306 (25.42%)	306 (25.42%)				0.890 (0.714-1110)	0.937 (0.741-1.184)
		T allele frequency	0.483	0.499		0.273			
		(TT+TG) vs. GG (dominant model)						0.853 (0.712-1.021)	0.924 (0.764-1.118)
		TT vs. (TG+GG) (recessive model)						1.000 (0.832-1.201)	0.990 (0.815-1.203)
	rs2069433	TT	1029 (85.47%)	1002 (83.22%)	0.198		0.089		
		CT	167 (13.87%)	188 (15.61%)				0.865 (0.690-1.084)	0.826 (0.652-1.045)
		CC	8 (0.66%)	14 (1.16%)				0.556 (0.232-1.332)	0.556 (0.219-1.412)
		C allele frequency	0.076	0.090		0.085			
		(CC+CT) vs. TT (dominant model)						0.844 (0.677-1.052)	0.808 (0.642-1.017)
		CC vs. (CT+TT) (recessive model)						0.569 (0.238-1.360)	0.571 (0.225-1.450)
CDK1	rs2448343	GG	790 (65.61%)	726 (60.30%)	**<0.0001**		**0.016**		
		AG	375 (31.15%)	419 (34.80%)				**0.822 (0.728-0.929)**	**0.832 (0.736-0.940)**
		AA	39 (3.24%)	59 (4.90%)				**0.607 (0.452-0.816)**	**0.614 (0.456-0.825)**
		A allele frequency	0.188	0.223		**0.003**			
		(AA+AG) vs. GG (dominant model)						**0.796 (0.708-0.895)**	**0.805 (0.715-0.905)**
		AA vs. (AG+GG) (recessive model)						**0.650 (0.485-0.870)**	**0.654 (0.488-0.876)**
	rs3213048	TT	483 (40.12%)	520 (43.19%)	0.215		0.080		
		CT	549 (45.60%)	534 (44.35%)				1.107 (0.932-1.314)	1.084 (0.903-1.300)
		CC	172 (14.29%)	150 (12.46%)				1.234 (0.960-1.587)	1.196 (0.916-1.562)
		C allele frequency	0.371	0.346		0.076			
		(CC+CT) vs. TT (dominant model)						1.135 (0.965-1.335)	1.108 (0.933-1.216)
		CC vs. (CT+TT) (recessive model)						1.171 (0.925-1.481)	1.147 (0.894-1.471)
	rs3213067	AA	862 (71.59%)	880 (73.09%)	0.423		0.272		
		GA	302 (25.08%)	294 (24.42%)				1.049 (0.871-1.263)	1.023 (0.840-1.245)
		GG	40 (3.32%)	30 (2.49%)				1.361 (0.840-2.205)	1.628 (0.953-2.779)
		G allele frequency	0.159	0.147		0.262			
		(GG+GA) vs. AA (dominant model)						1.078 (0.901-1.288)	1.070 (0.886-1.293)
		GG vs. (GA+AA) (recessive model)						1.345 (0.832-2.174)	1.618 (0.950-2.757)
	rs1871446	CC	946 (78.57%)	875 (72.67%)	**0.001**		**0.011**		
		TC	242 (20.10%)	288 (23.92%)				**0.777 (0.678-0.892)**	**0.784 (0.683-0.900)**
		TT	16 (1.33%)	41 (3.41%)				**0.361 (0.239-0.546)**	**0.343 (0.226-0.520)**
		T allele frequency	0.114	0.154		**<0.0001**			
		(TT+TC) vs. CC (dominant model)						0.725 (0.635-0.828)	0.728 (0.637-0.831)
		TT vs. (TC+CC) (recessive model)						0.382 (0.253-0.578)	0.362 (0.239-0.548)
	rs10711	GG	485 (40.28%)	481 (39.95%)	0.581		0.727		
		TG	527 (43.77%)	547 (45.43%)				0.955 (0.803-1.137)	0.890 (0.740-1.069)
		TT	192 (15.95%)	176 (14.62%)				1.082 (0.851-1.376)	1.019 (0.786-1.320)
		T allele frequency	0.378	0.373		0.721			
		(TT+TG) vs. GG (dominant model)						0.986 (0.838-1.161)	0.920 (0.774-1.093)
		TT vs. (TG+GG) (recessive model)						1.108 (0.887-1.384)	1.082 (0.851-1.377)
	rs1060343	GG	958 (79.57%)	989 (82.14%)	0.272		0.109		
		AG	235 (19.52%)	206 (17.11%)				1.177 (0.957-1.448)	1.158 (0.931-1.441)
		AA	11 (0.91%)	9 (0.75%)				1.261 (0.520-3.056)	0.991 (0.380-2.586)
		A allele frequency	0.107	0.093		0.113			
		(AA+AG) vs. GG (dominant model)						1.181 (0.964-1.447)	1.151 (0.929-1.427)
		AA vs. (AG+GG) (recessive model)						1.223 (0.505-2.963)	0.964 (0.370-2.514)

^*^ Two-sided chi-square test for difference in frequency distribution of genotypes between cases and controls.

^**^ Two-sided chi-square test for difference in frequency distribution of alleles between cases and controls.

^***^ Adjusted for age, BMI, age at menarche, age at first full-term pregnancy, menopause status, and family history of cancer in first-degree relatives.

Bold values indicate a statistical significance at 0.05 level.

### Associations of genotypes, haplotypes, and diplotypes with BC susceptibility

Two-sided chi-square test indicated significant differences both in allele frequencies and in genotype frequencies of rs2069429 (*CCNB1*), rs2448343 (*CDK1*), and rs1871446 (*CDK1*) (Table 1). In *CCNB1*, both univariate and multivariate logistic regression showed that rs2069429 (G>A) could increase the BC risk under recessive model (OR=2.352, 95% CI=1.480-3.737). In *CDK1*, the heterozygotes and minor allele homozygotes of both rs2448343 (G>A) and rs1871446 (C>T) could decrease the BC risk compared with the common homozygotes (Table 1). Multiple logistic regression analyses including these 2 SNPs in the full model was performed in order to select the more important SNPs associated with BC susceptibility. The result indicated that the statistical significance of rs2448343 disappeared with P=0.379 (OR=0.935, 95% CI=0.804-1.086), while the OR value for rs1871446 decreased a little (OR=0.763, 95% CI=0.644-0.904, P=0.002). The joint effects of these two protective loci in *CDK1* were also examined (Table 2). A dose-dependent effect of rs2448343 and rs1871446 in *CDK1* was observed with P_trend_=0.0002. In other words, women harboring two protective loci of rs2448343 and rs1871446 showed lower risk of BC than those harboring one protective locus. 

**Table 2:** Risk of breast cancer associated with the combination of 2 susceptible tSNPs in *CDK1*.

Genotype	Cases (%)	Controls (%)	OR (95%CI)		P value	aOR (95% CI)*	P value*
combinations of rs2448343 with rs1871446**							
0 susceptible locus	769 (63.87%)	689 (57.23%)					
1 susceptible locus	198 (16.45%)	223 (18.52%)		**0.796 (0.682-0.928)**	**0.0035**	**0.806 (0.691-0.941)**	**0.0063**
2 susceptible locus	237 (19.68%)	292 (24.25%)		**0.727 (0.631-0.838)**	**<0.0001**	**0.732 (0.636-0.844)**	**<0.0001**
Ptrend=0.0002							

^*^ Adjusted for age, BMI, age at menarche, age at first full-term pregnancy, menopause status, and family history of cancer in first-degree relatives.

^**^ Susceptible loci are defined as heterozygotes and homozygotes of minor allele of the 2 protective tSNPs.

Bold values indicate a statistical significance at 0.05 level.

 Given that age at menarche, number of births, age at first full-term pregnancy, family history of cancer and BMI were well known clinical risk factors of BC (Table S1), we then assessed whether the interactions between these clinical risk factors and the genetic variants would jointly affect BC susceptibility. We conducted stratified association analysis of genetic variants in *CDK1* by the above clinical risk factors. The result indicated that the association between AG or AA genotype of rs2448343 and decreased breast cancer risk was only significant among subjects with BMI<23 status (adjusted OR=0.579, 95% CI=0.465-0.720) (Table 3). Compared with women with AG or AA genotype of rs2448343 (adjusted OR=0.796, 95% CI=0.708-0.895) (Table 1) or BMI<23 status (adjusted OR=0.806, 95% CI=0.713-0.912) alone, women with both AG or AA genotype of rs2448343 and BMI<23 status had less BC susceptibility. No other significant association was observed in our study.

**Table 3:** Stratified analyses between rs2448343 of CDK1 and breast cancer risk by BMI status.

rs2448343	BMI<23		BMI≥23	
	case (%)/control (%)	aOR (95% CI)*	case (%)/control (%)	aOR (95% CI)*
GG	236 (69.41%)/222 (56.20%)	reference	554 (64.12%)/504 (62.30%)	reference
AG+AA	104 (30.59%)/173 (43.80%)	**0.579 (0.465-0.720)**	310 (35.88%)/305 (37.70%)	0.994 (0.815-1.094)

^*^ Adjusted for age, age at menarche, age at first full-term pregnancy, menopause status, and family history of cancer in first-degree relatives.

Bold values indicate a statistical significance at 0.05 level.

As haplotype and diplotype analysis may provide more power to detect association than single marker analyses alone [29], we also detected the associations of haplotypes and diplotypes in *CCNB1* and *CDK1* with breast cancer risk. The 4-SNP haplotype in *CCNB1* had no significant association with BC susceptibility (Table S3). However, in *CCNB1*, the 4-SNP haplotype pairs (diplotype) TAGT/TAGT (rs350104, rs2069429, rs164390, and rs2069433), which carried the minor allele homozygotes of the risk SNP rs2069429, could increase about 1.95-fold of BC risk (OR=1.947 95% CI=1.154-3.284, P=0.013, Table S3) compared with common diplotype TGTT/CGGT.

In *CDK1*, women harboring the 5-SNP haplotype ATATT (rs2448343, rs3213048, rs3213067, rs1871446, and rs10711), which carried 2 low-risk alleles of rs2448343 and rs1871446, had only 78.6% of BC risk compared with those harboring common haplotype GCACG (OR=0.786, 95% CI=0.646-0.957, P=0.017, Table S3). When it came to the diplotype, GCACG/GTGCT could increase the risk of BC (Table S4).

### Association of genotypes, haplotypes, and diplotypes with clinicopathological parameters

The associations of genotype, haplotype and diplotype with chinicopathological parameters (including ER status, PR status, Her2 status, tumor size, lymph node status, and clinical stage) were also examined in our study. 

In *CCNB1*, we found that patients harboring TT genotype of rs164390 (G>T) were less likely to have Her2-positive tumors (OR=0.573, 95% CI=0.379-0.868, P=0.009, shown in Table S5). Patients harboring the 4-SNP haplotypes CGGT and TAGT, which both carried the major allele of rs164390, were more likely to have Her2-positive BC compared with patients harboring common haplotype TGTT (CGGT: OR=1.346, 95% CI=1.039-1.744, P=0.025; TAGT: OR=1.424, 95% CI=1.059-1.391, P=0.019; Table S6). Diplotype analysis indicated that TGTT/TGTT, which carried the minor allele homozygotes of rs164390, was more likely to develop Her2-negative BC (OR=0.577, 95% CI=0.402-0.828, P=0.003, Table S7).

In *CDK1*, patients with heterozygotes of rs2448343 (G>A) were less likely to develop tumors with PR-negative status (OR=0.710, 95% CI=0.521-0.969, P=0.031), while patients with CC genotype of rs3213048 (T>C) or AG genotype of rs3213067 (A>G) were more likely to have PR-negative tumors compared with those carrying corresponding common genotypes (rs3213048: OR=1.619, 95% CI=1.068-2.455, P=0.023; rs3213067: OR=1.410, 95% CI=1.031-1.930, P=0.032) (Table S8). Haplotype analysis also indicated that patients with GTACG, ATACT, and GTATT haplotype were more likely to develop PR-positive BC compared with those carrying common haplotype GCACG (Table S9). Besides, compared to women carrying common diplotype GCACG/GTACG, patients harboring GTACG/ATATT diplotype were more likely to have less aggressive tumors such as negative lymph nodes (OR=0.455, 95%CI=0.274-0.753, P=0.002), size≤2cm tumors (OR=0.613, 95% CI=0.385-0.975, P=0.039) and clinical stage 0-I tumors (OR=0.390, 95% CI=0.227-0.671, P=0.0007). Furthermore, the GTGCT/ATACT diplotype was found to be associated with ER-positive tumors (OR=0.121, 95% CI=0.029-0.506, P=0.004) and PR-positive tumors (OR=0.224, 95% CI=0.078-0.638, P=0.005).

### Associations of genotypes, haplotypes, and diplotypes with event-free survival

First of all, the association of the clinicopathological parameters with event-free survival was analyzed. As expected, aggressive clinicopathological parameters, such as PR-negative status, Her2-positive status, tumor size >2cm, lymph node metastasis and clinical stage II-IV, were all associated with worse survival in both Kaplan-Meier log-rank analysis and the Cox’s proportional hazard model analysis (Table 4). 

**Table 4:** Association analysis of the clinicopathological parameters in relation to event-free survival of breast cancer patients (n=1005).

Parameter	No.	No_event_ (%)	Log-rank P value	HR (95%CI)	P value	aHR (95%CI)*	P value*
age							
≤50y	521	86 (16.51%)	0.173				
>50y	484	67 (13.84%)		0.801 (0.582-1.103)	0.174	0.809 (0.582-1.126)	0.210
ER							
positive	556	68 (12.23%)	0.153				
negative	225	37 (16.44%)		1.342 (0.896-2.011)	0.154	1.468 (0.977-2.207)	0.065
PR							
positive	511	57 (11.15%)	**0.006**				
negative	266	48 (18.05%)		**1.716 (1.167-2.524)**	**0.006**	**1.898 (1.287-2.798)**	**0.001**
Her2							
negative	576	68 (11.81%)	**0.038**				
positive	202	37 (18.32%)		**1.526 (1.020-2.281)**	**0.039**	**1.519 (1.016-2.271)**	**0.042**
Lymoph node metastasis							
negative	406	49 (12.07)	**<0.0001**				
positive	279	82 (29.39%)		**2.785 (1.953-3.973)**	**<0.0001**	**2.425 (1.686-3.486)**	**<0.0001**
Size							
<=2cm	338	31 (9.17%)	**0.0008**				
>2cm	464	84 (18.10%)		**1.998 (1.322-3.019)**	**0.001**	**1.913 (1.265-2.892)**	**0.002**
Clinical stage							
0-I	113	9 (7.96%)	**0.004**				
II-IV	581	107 (18.42%)		**2.590 (1.311-5.114)**	**0.006**	**2.278 (1.150-4.510)**	**0.018**

^*^ Adjusted for age, BMI, age at menarche, age at first full-term pregnancy, menopause status, and family history of cancer in first-degree relatives.

Bold values indicate a statistical significance at 0.05 level.

**Figure 2:**
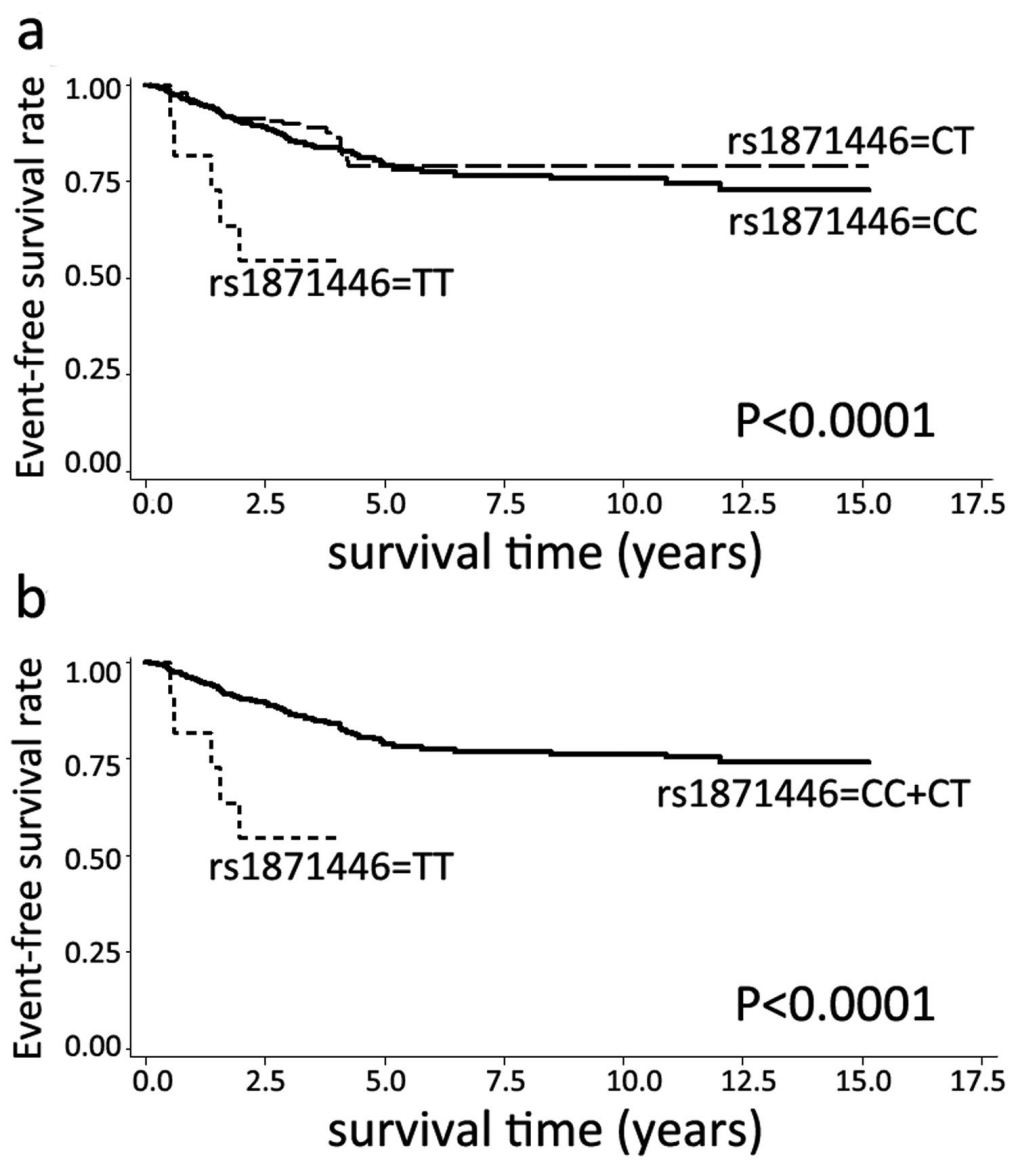
Kaplan-Meier estimates of event-free survival according to rs1871446 genotypes.

**Figure 3:**
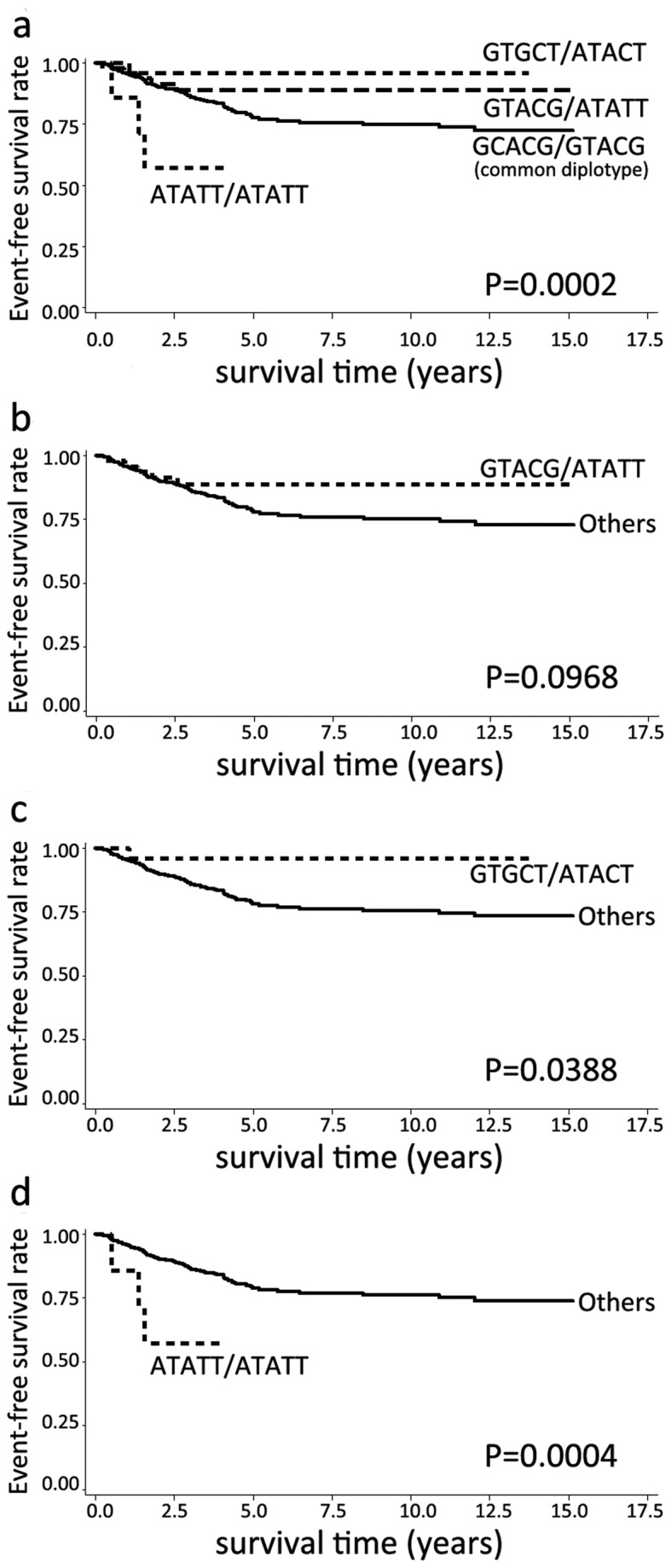
Kaplan-Meier estimates of event-free survival according to CDK1 diplotypes.

Then, we analyzed the associations of genotypes, haplotypes and diplotypes with event-free survival. In *CCNB1* gene, no tSNPs, haplotypes and diplotypes were associated with event-free survival (data not shown). In *CDK1* gene, the minor allele homozygotes of rs1871446 (C>T) were correlated with an unfavorable event-free survival (HR=4.323, 95% CI=1.763-10.599, P=0.001, Table 5). Figure 2A showed the survival curve of rs1871446 genotypes, and Figure 2B demonstrated the survival curve of rs1871446 under recessive model. Patients harboring the diplotype ATATT/ATATT, which carried the minor allele homozygotes of rs1871446, had a worse event-free survival compared with the common diplotype GCACG/GTACG (HR=3.022, 95% CI=1.309-6.975, P=0.009, Table 5). Diplotype analysis also indicated that patients with GTACG/ATATT, which has significant association with less aggressive tumors previously (including negative lymph nodes, size ≤2cm tumors, and clinical stage 0-I tumors), had a favorable event-free survival when compared with common diplotype (HR=0.471, 95% CI=0.242-0.917, P=0.027, Table 5). In addition, patients harboring diplotype GTGCT/ATACT, which was associated with ER-positive and PR-positive tumors, also had a better event-free survival (HR=0.205, 95% CI=0.050-0.838, P=0.027, Table 5). The survival curves of these three diplotypes were shown in Figure 3.

**Table 5:** Association analysis of the selected tSNPs, diplotypes in CDK1 in relation to event-free survival of breast cancer patients (n=1005).

Parameter	No.	No_event_ (%)	Log-rank P value	HR (95%CI)	P value	aHR (95%CI)*	P value*
*CDK1 tSNPs*							
rs2448343							
GG	665	107 (16.09%)	0.241				
AG	305	39 (12.79%)		0.762 (0.528-1.100)	0.147	0.760 (0.523-1.106)	0.152
AA	35	7 (20.00%)		1.293 (0.602-2.778)	0.510	1.262 (0.585-2.719)	0.553
(AA+AG) vs. GG (dominant model)				0.813 (0.576-1.149)	0.241	0.811 (0.570-1.153)	0.243
AA vs. (AG+GG) (recessive model)				1.399 (0.655-2.987)	0.386	1.370 (0.640-2.9330	0.417
rs3213048							
TT	404	53 (13.12%)	0.072				
CT	460	83 (18.04%)		**1.429 (1.013-2.018)**	**0.042**	**1.450 (1.019-2.063)**	**0.039**
CC	141	17 (12.06%)		0.954 (0.552-1.647)	0.865	0.863 (0.485-1.536)	0.617
(CC+CT) vs. TT (dominant model)				1.318 (0.944-1.839)	0.104	1.307 (0.929-1.839)	0.124
CC vs. (CT+TT) (recessive model)				0.779 (0.471-1.290)	0.332	0.701 (0.410-1.197)	0.193
rs3213067							
AA	715	109 (15.24%)	0.963				
GA	254	39 (15.35%)		1.030 (0.715-1.485)	0.873	1.047 (0.718-1.525)	0.813
GG	36	5 (13.89%)		0.909 (0.371-2.229)	0.835	0.970 (0.395-2.381)	0.946
(GG+GA) vs. AA (dominant model)				1.015 (0.715-1.440)	0.934	1.037 (0.724-1.485)	0.844
GG vs. (GA+AA) (recessive model)				0.902 (0.370-2.200)	0.821	0.958 (0.392-2.342)	0.926
rs1871446							
CC	789	121 (15.34%)	**0.001**				
TC	205	27 (13.17%)		0.831 (0.547-1.261)	0.384	0.777 (0.507-1.190)	0.246
TT	11	5 (45.45%)		**4.323 (1.763-10.599)**	**0.001**	**5.795 (2.317-14.492)**	**0.0002**
(TT+TC) vs. CC (dominant model)				0.951 (0.644-1.404)	0.064	0.905 (0.608-1.348)	0.623
TT vs. (TC+CC) (recessive model)				**4.482 (1.834-10.954)**	**0.001**	**5.087 (2.071-12.497)**	**0.0004**
rs10711							
GG	408	59 (14.46%)	0.981				
TG	441	70 (15.87%)		1.035 (0.731-1.465)	0.846	1.098 (0.768-1.570)	0.608
TT	156	24 (15.38%)		1.011 (0.628-1.627)	0.965	1.193 (0.734-1.940)	0.477
(TT+TG) vs. GG (dominant model)				1.029 (0.742-1.426)	0.865	1.121 (0.802-1.568)	0.503
TT vs. (TG+GG) (recessive model)				0.992 (0.641-1.535)	0.972	1.136 (0.727-1.774)	0.577
rs1060373							
GG	807	124 (15.37%)	0.923				
AG	192	28 (14.58%)		0.974 (0.646-1.468)	0.900	0.944 (0.621-1.434)	0.787
AA	6	1 (16.67%)		1.460 (0.204-10.431)	0.706	1.427 (0.199-10.245)	0.724
(AA+AG) vs. GG (dominant model)				0.985 (0.658-1.476)	0.943	0.955 (0.632-1.443)	0.828
AA vs. (AG+GG) (recessive model)				1.467 (0.206-10.468)	0.702	1.443 (0.201-10.344)	0.715
*CDK1 diplotypes*							
GCACG/GTACG	181	33 (18.23%)	\				
GCACG/GCACG	113	13 (11.50%)	\	0.601 (0.316-1.143)	0.121	**0.496 (0.250-0.986)**	**0.046**
GCACG/ATATT	98	22 (22.45%)	\	1.188 (0.811-1.741)	0.377	1.194 (0.814-1.752)	0.345
GTACG/GTGCT	73	10 (13.70%)	\	0.663 (0.402-1.094)	0.108	0.710 (0.430-1.172)	0.180
GCACG/GTGCT	68	8 (11.76%)	\	0.598 (0.276-1.295)	0.192	0.664 (0.305-1.444)	0.301
GTACG/GTACG	66	8 (12.12%)	\	0.548 (0.253-1.187)	0.127	0.556 (0.255-1.213)	0.140
GTACG/ATATT	51	5 (9.80%)	\	**0.471 (0.242-0.917)**	**0.027**	**0.470 (0.241-0.915)**	**0.026**
GCACG/ATACT	47	7 (14.89%)	\	0.678 (0.380-1.208)	0.187	0.644 (0.361-1.151)	0.138
GTACG/ATACT	42	9 (21.43%)	\	1.139 (0.676-1.918)	0.625	1.011 (0.599-1.705)	0.968
GTGCT/ATATT	30	4 (13.33%)	\	0.715 (0.343-1.490)	0.371	0.641 (0.307-1.333)	0.236
GTGCT/ATACT	27	3 (11.11%)	\	0.524 (0.227-1.210)	0.131	0.501 (0.217-1.157)	0.106
GTGCT/GTGCT	25	1 (4.00%)	\	**0.205 (0.050-0.838)**	**0.027**	**0.192 (0.047-0.785)**	**0.022**
ATATT/ATACT	13	2 (15.38%)	\	0.800 (0.292-2.196)	0.665	0.805 (0.293-2.213)	0.675
ATATT/ATATT	7	3 (42.86%)	\	**3.022 (1.309-6.975)**	**0.009**	**3.988 (1.712-9.290)**	**0.001**
else	164	25 (15.24%)	\	0.810 (0.560-1.171)	0.262	0.770 (0.532-1.114)	0.165

^*^ Adjusted for age, BMI, age at menarche, age at first full-term pregnancy, menopause status, and family history of cancer in first-degree relatives.

Bold values indicate a statistical significance at 0.05 level.

## Discussion

Chinese Han population is the largest ethnic group and constitutes about 92% of the population of the People’s Republic of China. Many studies investigated the associations between SNPs and breast cancer susceptibility among Chinese Han population. Some SNPs in DNA repair related genes, such as *APE1*, *XRCC1*, *ERCC1* and *XPF* [30-32] were found to be associated with breast cancer susceptibility in Chinese Han population, so did some SNPs in cell-cycle genes such as *CCNE1* and *CDK2* [33]. Also, some meta-analysis studies found that SNPs in genes *MDR*, *MTR*, *SLC4A7*, *ATR* and *CHEK1* were significantly associated with breast cancer susceptibility [34-37]. However, as far as we know, this is the first study to comprehensively evaluate the association of germline variation in *CCNB1* and *CDK1*, two essential centrosome-regulating genes in cell cycle, with BC risk, progression and survival in Chinese Han population.

*CCNB1* and *CDK1* genes encode CyclinB1 and CDK1, which are two critical proteins and interact with each other in cell cycle. Accumulating evidence demonstrated that both the CyclinB1 and CDK1 overexpression could contribute to cancer risk and progressions [14,15,20]. In this study, we hypothesized that the genetic variation in *CCNB1* and *CDK1* had great impact on susceptibility, progression and survival of breast cancer. With a case-control study including 1204 breast cancer patients and 1204 age-matched controls, we genotyped 10 tSNPs of these two genes.

For *CCNB1*, 4 tSNPs including rs350104, rs2069429, rs164390 and rs2069433 were analyzed, and these 4 SNPs formed a 4-SNP haplotype block according to our control population data. The result revealed that rs2069429 was significantly associated with high BC susceptibility under recessive model. Diplotype analysis in *CCNB1* showed that the diplotype TAGT/TAGT, which carried the minor allele homozygotes of rs2069429, was more likely to have BC than the common diplotypes. The SNP rs2069429 is located in 0.1 kbp upstream of *CCNB1* and may be the regulatory region of the *CCNB1* transcription. Notably, Hui Cai and colleagues genotyped 3 tagging SNPs of *CCNB1* in 1449 newly diagnosed endometrial cases from Shanghai Cancer Registry in China, and found that rs2069433 was related to a reduction in endometrial cancer risk [18], however, no significant association was observed between rs2069433 and breast carcinoma susceptibility in our study. Four SNPs of *CCNB1* (rs352626, rs350104, rs2069429, rs164390) were genotyped by H Ma and colleagues in 828 non-small cell lung cancer cases, and rs2069429 was found to be associated with NSCLC survival with a log-rank P<0.1 under recessive model [19]. In our survival analysis, no significant association in *CCNB1* was observed. The difference between our result and H Ma’s result can be explained as follows. Firstly, our cases were 1204 breast carcinomas while their cases were 828 NSCLC patients. Secondly, a two-sided P value<0.05 was considered statistically significant in our study instead of P<0.1. Our results also demonstrated that TT of rs164390 in *CCNB1* was associated with Her2-negative tumors, consistent with the result that the haplotypes of CGGT and TAGT were associated with Her2-positive tumors and diplotype TGTT/TGTT were associated with Her2-negative tumors. No previous research has analyzed the association of rs164390 with Her2 status in any tumor. The SNP rs164390 is located in the 5’-UTR of the gene *CCNB1*. 5’-UTR has been mainly implicated in translational control, affecting all post-transcriptional stages such as mRNA stability, folding, and interactions with the ribosomal machinery [38,39]. Nevertheless, there is also the possibility that rs2069429 and rs164390 are only the tags of the causal variants. Therefore, fine-mapping to this region and further functional experiments are warranted in order to determine whether these tSNPs are the causal variants.

For *CDK1*, 6 tSNPs were genotyped, being rs2448343, rs3213048, rs3213067, rs1871446, rs10711 and rs1060373. The first five SNPs were constructed as a 5-SNP haplotype block in our control population. We found that the minor alleles in rs2448343 and rs1871446 were significantly associated with low BC risk. Multiple logistic regression analysis including these 2 SNPs in the full model indicated that rs1871446 had a stronger effect on reducing BC risk than rs2448343. These 2 SNP showed a dose-dependent effect on the BC risk (P_trend_=0.0002). Haplotype analysis also indicated that ATATT, which contained two minor alleles of both rs2448343 and rs1871446, showed significant association with low BC susceptibility. For the gene-gene and gene-environment interaction analysis, some previous studies used the method of multifactor dimensionality reduction (MDR) [40,41]. The method of MDR is a non-parametric, genetic model-free method for overcoming the limitation of small sample size. As we had enough samples in our study, the stratified association analysis was used to test the interaction between tSNPs and clinical parameters on BC risk. We observed a significant joint effect of rs2448343 and BMI status on BC susceptibility: compared with women with AG or AA genotype of rs2448343 or BMI<23 status alone, women with both AG or AA genotype of rs2448343 and BMI<23 status had less BC susceptibility. The SNP rs2448343 is located in intron region of *CDK1*, which may influence the disease risk by affecting mRNA expression levels, alternative splicing, mRNA structure and mRNA stability [42,43]. The SNP rs1871446 is located in the 3’-UTR of *CDK1*, which is essential in mRNA stability [44,45] and localization [46]. 3’-UTR may also be the binding site of miRNA. Our result also indicated that three closely located SNPs, rs2448343, rs3213048 and rs3213067, were significantly associated with tumor’s PR status: the heterozygotes of rs2448343 were associated with PR-positive tumors, while minor allele homozygotes of rs3213048 and heterozygotes of rs3213067 were associated with PR-negative BC tumors. Haplotype analysis indicated that patients with GTACG, ATACT, and GTATT were more likely to develop PR-positive BC tumors compared with common haplotype GCACG. Besides, diplotype analysis indicated that GTACG/ATATT were associated with less aggressive tumors such as negative lymph nodes, size ≤2cm tumors, and clinical stage 0-I tumors; while GTGCT/ATACT was found to be associated with less aggressive tumors such as ER positive-tumors or PR-positive tumors compared to the common diplotype GCACG/GTACG, which is consistent with the survival analysis results in which the patients harboring diplotypes of GTACG/ATATT and GTGCT/ATACT had a favorable event-free survival. In survival analysis, H Ma and colleague genotyped 3 SNPs of *CDK1* including rs2127355, rs2170006 and rs1871446, but no significant association between these SNPs and NSCLC survival was observed [19]. In our study, the minor allele homozygotes TT of rs1871446 had an unfavorable breast carcinoma survival under recessive model. Diplotype analysis also proved that ATATT/ATATT, which carried the minor allele homozygotes of rs1871446, had a negative impact on event-free survival.

In summary, in *CCNB1*, rs2069429 and diplotype TAGT/TAGT were associated with increased BC susceptibility. In *CDK1*, rs2448343 and rs1871446 were associated with decreased BC risk, so was the *CDK1* haplotype ATATT. These two SNPs also showed a dose-dependent effect on the BC susceptibility. Notably, the minor allele homozygote of rs1871446 was associated with unfavorable event-free survival, so was the diplotype ATATT/ATATT in *CDK1*. Nevertheless, these results need to be verified in other populations. Functional studies are also needed to determine how these SNPs influence the BC susceptibility and event-free survival. 

## Supporting Information

Table S1 **The characteristics data of the cases and controls.**
(DOC)

Table S2 **The Hardy-Weinberg equilibrium of the 10 tSNPs.**
(DOC)

Table S3 **The association between the haplotypes and breast cancer risk.**
(DOC)

Table S4 **The association between the diplotypes and breast cancer risk.**
(DOC)

Table S5 **The association analysis of the genotype in *CCNB1* in relation to Her2 status.**
(DOC)

Table S6 **The association between the haplotypes in *CCNB1* and Her2 status.**
(DOC)

Table S7 **The association between the diplotype in *CCNB1* and Her2 status.**
(DOC)

Table S8 **The association between the haplotypes in *CDK1* and PR status.**
(DOC)

Table S9 **The association between the haplotypes in *CDK1* and PR status.**
(DOC)
