# Supplementary material for: Association Study of Germline Variants in CCNB1 and CDK1 with Breast Cancer Susceptibility, Progression, and Survival among Chinese Han Women
Source: PLoS One. 2013 Dec 27;8(12):e84489. doi: 10.1371/journal.pone.0084489 (PMC3873991; doi:10.1371/journal.pone.0084489)
Supplement: Table S1 — The characteristics data of the cases and controls. (DOC) [file pone.0084489.s001.doc]

Table S1. The characteristics data of the cases and controls.

| variable | cases, n=1204 | controls, n=1204 | P value |
| --- | --- | --- | --- |
| age, years (mean±SD) | 48.981±10.077 | 48.665±9.857 | 0.437 |
| BMI (mean±SD) | 24.581±3.175 | 24.476±3.555 | 0.447 |
| age at menarche, years (mean±SD) | 14.546±1.815 | 15.035±1.878 | **<0.0001** |
| age at menopause, years (mean±SD) | 48.998±4.160 | 49.154±3.979 | 0.554 |
| age at first full-time pregnancy, years (mean±SD) | 26.133±2.983 | 25.396±2.740 | **<0.0001** |
| birthnumber (mean±SD) | 2.055±1.172 | 2.518±1.292 | **<0.0001** |
| age | | | |
| <50y | 632 (52.49%) | 674 (55.98%) | 0.086 |
| ≥50y | 572 (47.51%) | 530 (44.02%) |
| BMI | | | |
| <23 | 340 (28.24%) | 395 (32.81%) | **0.015** |
| ≥23 | 864 (71.76%) | 809 (67.19%) |
| menarche | | | |
| ≥12-year-old | 1183 (98.26%) | 1190 (98.84%) | 0.233 |
| <12-year-old | 21 (1.74%) | 14 (1.16%) |
| Birth number | | | |
| ≥1 | 1182 (98.17%) | 1181 (98.09%) | 0.880 |
| 0 | 22 (1.83%) | 23 (1.91%) |
| breastfeeding | | | |
| <6 months | 999 (89.12%) | 944 (78.41%) | **<0.0001** |
| ≥6 months | 122 (10.88%) | 260 (21.59%) |
| menopause | | | |
| post | 576 (47.84%) | 534 (44.35%) | 0.086 |
| pre | 628 (52.16%) | 670 (55.65%) |
| Family history | | | |
| no | 949 (78.82%) | 988 (82.06%) | **0.045** |
| yes | 255 (21.18%) | 216 (17.94%) |
| estrogen receptor (ER) | | | |
| positive | 645 (53.57%) |  |  |
| negative | 259 (21.51%) |  |
| missing data | 300 (24.92%) |  |
| progesterone receptor (PR) | | | |
| positive | 596 (49.50%) |  |  |
| negative | 304 (25.25%) |  |
| missing data | 304 (25.25%) |  |
| Her2 | | | |
| positive | 240 (19.93%) |  |  |
| negative | 661 (54.90%) |  |
| missing data | 303 (25.17%) |  |
| tumor size in cm | | | |
| ≤2cm | 389 (32.31%) |  |  |
| >2cm | 537 (44.60%) |  |
| missing data | 278 (23.09%) |  |
| lymph node metastasis | | | |
| negative | 467 (38.78%) |  |  |
| positive | 336 (27.91%) |  |
| missing data | 401 (33.31%) |  |
| Clinical stage at diagnosis | | | |
| 0-I | 136 (11.30%) |  |  |
| I-II | 691 (57.39%) |  |
| missing data | 377 (31.31%) |  |
