# Supplementary material for: Association Study of Germline Variants in CCNB1 and CDK1 with Breast Cancer Susceptibility, Progression, and Survival among Chinese Han Women
Source: PLoS One. 2013 Dec 27;8(12):e84489. doi: 10.1371/journal.pone.0084489 (PMC3873991; doi:10.1371/journal.pone.0084489)
Supplement: Table S2 — The Hardy-Weinberg equilibrium of the 10 tSNPs. (DOC) [file pone.0084489.s002.doc]

Table S2. The Hardy-Weinberg equilibrium of the 10 tSNPs.

| SNP | Gene | p | HWE |
| --- | --- | --- | --- |
| rs350104 | CCNB1 | 0.499 | Yes |
| rs2069429 | CCNB2 | 0.740 | Yes |
| rs164390 | CCNB3 | 0.498 | Yes |
| rs2069433 | CCNB4 | 0.154 | Yes |
| rs2448343 | CDK1 | 0.930 | Yes |
| rs3213048 | CDK2 | 0.492 | Yes |
| rs3213067 | CDK3 | 0.359 | Yes |
| rs1871446 | CDK4 | 0.410 | Yes |
| rs10711 | CDK5 | 0.327 | Yes |
| rs1060373 | CDK6 | 0.736 | Yes |
