# Supplementary material for: Association Study of Germline Variants in CCNB1 and CDK1 with Breast Cancer Susceptibility, Progression, and Survival among Chinese Han Women
Source: PLoS One. 2013 Dec 27;8(12):e84489. doi: 10.1371/journal.pone.0084489 (PMC3873991; doi:10.1371/journal.pone.0084489)
Supplement: Table S3 — The association between the haplotypes and breast cancer risk. (DOC) [file pone.0084489.s003.doc]

Table S3. The association between the haplotypes and breast cancer risk.

| GENE | Haplotype | Cases (%) | Controls (%) | OR (95% CI) | P value | aOR (95%CI) | P value |
| --- | --- | --- | --- | --- | --- | --- | --- |
| CCNB1 | TGTT | 954 (39.62%) | 977 (40.57%) |  |  |  |  |
|  | CGGT | 691 (28.70%) | 709 (29.44%) | 0.998 (0.870-1.145) | 0.979 | 0.993 (0.865-1.140) | 0.919 |
|  | TAGT | 412 (17.11%) | 367 (15.24%) | 1.150 (0.973-1.358) | 0.101 | 1.149 (0.972-1.358) | 0.104 |
|  | TGTC | 178 (7.39%) | 213 (8.85%) | 0.856 (0.688-1.064) | 0.162 | 0.841 (0.676-1.047) | 0.121 |
|  | TGGT | 131 (5.44%) | 125 (5.19%) | 1.073 (0.827-1.393) | 0.595 | 1.097 (0.844-1.425) | 0.489 |
|  | else | 42 (1.74%) | 17 (0.71%) | 2.528 (1.429-4.473) | 0.001 | 2.587 (1.459-4.586) | 0.001 |
|  |  |  |  |  |  |  |  |
| CDK1 | GCACG | 800 (33.22%) | 797 (33.10%) |  |  |  |  |
|  | GTACG | 617 (25.63%) | 664 (27.57%) | 0.926 (0.799-1.072) | 0.304 | 0.918 (0.792-1.064) | 0.257 |
|  | GTGCT | 336 (13.95%) | 332 (13.78%) | 1.008 (0.842-1.208) | 0.929 | 1.002 (0.836-1.201) | 0.981 |
|  | ATATT | 236 (9.80%) | 299 (12.42%) | **0.786 (0.646-0.957)** | **0.017** | **0.786 (0.645-0.958)** | **0.017** |
|  | ATACT | 178 (7.39%) | 194 (8.06%) | 0.914 (0.729-1.146) | 0.435 | 0.927 (0.739-1.162) | 0.509 |
|  | GTATT | 28 (1.16%) | 30 (1.25%) | 0.930 (0.550-1.571) | 0.786 | 0.950 (0.561-1.607) | 0.848 |
|  | else | 213 (8.85%) | 92 (3.82%) | 2.306 (1.772-3.001) | <0.0001 | 2.296 (1.763-2.990) | 0.0002 |
