# Supplementary material for: Association Study of Germline Variants in CCNB1 and CDK1 with Breast Cancer Susceptibility, Progression, and Survival among Chinese Han Women
Source: PLoS One. 2013 Dec 27;8(12):e84489. doi: 10.1371/journal.pone.0084489 (PMC3873991; doi:10.1371/journal.pone.0084489)
Supplement: Table S4 — The association between the diplotypes and breast cancer risk. (DOC) [file pone.0084489.s004.doc]

Table S4. The association between the diplotypes and breast cancer risk.

| Gene | Diplotype | Cases (%) | Controls (%) | OR (95%CI) | P value | aOR (95%CI) | P value |
| --- | --- | --- | --- | --- | --- | --- | --- |
| CCNB1 | TGTT/CGGT | 252 (20.93%) | 292 (24.25%) |  |  |  |  |
|  | TGTT/TGTT | 220 (18.27%) | 207 (17.19%) | 1.232 (0.955-1.587) | 0.108 | 1.145 (0.874-1.501) | 0.326 |
|  | TGTT/TAGT | 144 (11.96%) | 140 (11.63%) | 1.192 (0.894-1.588) | 0.231 | 1.138 (0.838-1.546) | 0.406 |
|  | CGGT/TAGT | 120 (9.97%) | 115 (9.55%) | 1.209 (0.890-1.642) | 0.224 | 1.064 (0.769-1.471) | 0.709 |
|  | CGGT/CGGT | 108 (8.97%) | 99 (8.22%) | 1.264 (0.917-1.742) | 0.152 | 1.051 (0.747-1.478) | 0.776 |
|  | TGTT/TGTC | 67 (5.56%) | 81 (6.73%) | 0.958 (0.665-1.381) | 0.820 | 0.869 (0.594-1.271) | 0.469 |
|  | CGGT/TGTC | 60 (4.98%) | 64 (5.32%) | 1.086 (0.735-1.605) | 0.678 | 0.952 (0.634-1.431) | 0.813 |
|  | TGTT/TGGT | 47 (3.90%) | 46 (3.82%) | 1.184 (0.762-1.838) | 0.452 | 1.268 (0.793-2.028) | 0.322 |
|  | CGGT/TGGT | 39 (3.24%) | 36 (2.99%) | 1.255 (0.774-2.035) | 0.366 | 1.163 (0.698-1.938) | 0.562 |
|  | TAGT/TAGT | 42 (3.49%) | 25 (2.08%) | **1.947 (1.154-3.284)** | **0.013** | **1.781 (1.031-3.077)** | **0.039** |
|  | TAGT/TGTC | 29 (2.41%) | 32 (2.66%) | 1.050 (0.618-1.784) | 0.857 | 0.994 (0.571-1.732) | 0.983 |
|  | TAGT/TGGT | 23 (1.91%) | 28 (2.33%) | 0.952 (0.535-1.694) | 0.867 | 0.940 (0.508-1.740) | 0.844 |
|  | else | 53 (4.40%) | 39 (3.24%) | 1.575 (1.008-2.461) | 0.046 | 1.531 (0.956-2.453) | 0.077 |
| CDK1 | GCACG/GTACG | 209 (17.36%) | 238 (19.77%) |  |  |  |  |
|  | GCACG/GCACG | 140 (11.63%) | 136 (11.30%) | 1.172 (0.948-1.450) | 0.143 | 1.179 (0.952-1.459) | 0.131 |
|  | GCACG/GTGCT | 116 (9.63%) | 97 (8.06%) | **1.362 (1.080-1.717)** | **0.009** | **1.353 (1.072-1.707)** | **0.011** |
|  | GTACG/GTACG | 82 (6.81%) | 83 (6.89%) | 1.125 (0.874-1.448) | 0.361 | 1.096 (0.850-1.413) | 0.480 |
|  | GTACG/GTGCT | 78 (6.48%) | 99 (8.22%) | 0.897 (0.700-1.149) | 0.390 | 0.876 (0.683-1.123) | 0.296 |
|  | GCACG/ATATT | 76 (6.31%) | 101 (8.39%) | 0.857 (0.669-1.098) | 0.222 | 0.848 (0.661-1.087) | 0.193 |
|  | GTACG/ATATT | 65 (5.40%) | 79 (6.56%) | 0.937 (0.718-1.223) | 0.632 | 0.948 (0.725-1.239) | 0.696 |
|  | GTACG/ATACT | 58 (4.82%) | 61 (5.07%) | 1.083 (0.813-1.441) | 0.586 | 1.089 (0.817-1.452) | 0.559 |
|  | GCACG/ATACT | 54 (4.49%) | 65 (5.40%) | 0.946 (0.710-1.261) | 0.705 | 0.956 (0.716-1.275) | 0.758 |
|  | GTGCT/ATATT | 36 (2.99%) | 43 (3.57%) | 0.953 (0.679-1.339) | 0.783 | 0.956 (0.680-1.344) | 0.794 |
|  | GTGCT/GTGCT | 30 (2.49%) | 23 (1.91%) | 1.485 (0.990-2.229) | 0.056 | 1.463 (0.973-2.200) | 0.067 |
|  | GTGCT/ATACT | 29 (2.41%) | 27 (2.24%) | 1.223 (0.825-1.812) | 0.315 | 1.255 (0.846-1.863) | 0.259 |
|  | ATATT/ATACT | 16 (1.33%) | 18 (1.50%) | 1.012 (0.618-1.659) | 0.962 | 1.013 (0.617-1.662) | 0.960 |
|  | ATATT/ATATT | 10 (0.83%) | 18 (1.50%) | 0.633 (0.361-1.110) | 0.111 | 0.625 (0.355-1.101) | 0.104 |
|  | else | 205 (17.03%) | 116 (9.63%) | 2.012 (1.635-2.477) | <0.0001 | 2.021 (1.640-2.490) | <0.0001 |
