# Supplementary material for: Association Study of Germline Variants in CCNB1 and CDK1 with Breast Cancer Susceptibility, Progression, and Survival among Chinese Han Women
Source: PLoS One. 2013 Dec 27;8(12):e84489. doi: 10.1371/journal.pone.0084489 (PMC3873991; doi:10.1371/journal.pone.0084489)
Supplement: Table S5 — The association analysis of the genotype in CCNB1 in relation to Her2 status. (DOC) [file pone.0084489.s005.doc]

Table S5. The association analysis of the genotype in *CCNB1* in relation to Her2 status.

| Gene | SNP | Her2 | | | | | | |
| --- | --- | --- | --- | --- | --- | --- | --- | --- |
|  |  | χ^2^ | | | Logistical Regression | | | |
|  |  | Negative | Positive | P value | OR (95%CI) | P value | aOR (95%CI) | P value |
| CCNB1 | rs350104(T>C) |  |  |  |  |  |  |  |
|  | TT | 350 (52.95%) | 110 (45.83%) | 0.166 |  |  |  |  |
|  | CT | 253 (38.28%) | 105 (43.75%) |  | 1.321 (0.966-1.805) | 0.081 | 1.298 (0.947-1.780) | 0.105 |
|  | CC | 58 (8.77%) | 25 (10.42%) |  | 1.371 (0.819-2.297) | 0.229 | 1.356 (0.807-2.281) | 0.250 |
|  | rs2069429(G>A) |  |  |  |  |  |  |  |
|  | GG | 454 (68.68%) | 154 (64.17%) | 0.343 |  |  |  |  |
|  | AG | 173 (26.177%) | 69 (28.75%) |  | 1.176 (0.842-1.641) | 0.341 | 1.188 (0.848-1.664) | 0.317 |
|  | AA | 34 (5.14%) | 17 (7.08%) |  | 1.474 (0.801-2.714) | 0.213 | 1.457 (0.789-2.691) | 0.229 |
|  | rs164390(G>T) |  |  |  |  |  |  |  |
|  | GG | 176 (26.63%) | 82 (34.17%) | **0.029** |  |  |  |  |
|  | TG | 309 (46.75%) | 111 (46.25%) |  | 0.771 (0.549-1.083) | 0.134 | 0.783 (0.556-1.102) | 0.161 |
|  | TT | 176 (26.63%) | 47 (19.58%) |  | **0.573 (0.379-0.868)** | **0.009** | **0.579 (0.382-0.880)** | **0.010** |
|  | rs2069433(T>C) |  |  |  |  |  |  |  |
|  | TT | 562 (85.02%) | 207 (86.25%) | 0.724 |  |  |  |  |
|  | CT | 93 (14.07%) | 32 (13.33%) |  | 0.934 (0.606-1.439) | 0.758 | 0.933 (0.604-1.441) | 0.754 |
|  | CC | 6 (0.91%) | 1 (0.42%) |  | 0.453 (0.054-3.781) | 0.464 | 0.458 (0.054-3.857) | 0.473 |
