# Supplementary material for: Association Study of Germline Variants in CCNB1 and CDK1 with Breast Cancer Susceptibility, Progression, and Survival among Chinese Han Women
Source: PLoS One. 2013 Dec 27;8(12):e84489. doi: 10.1371/journal.pone.0084489 (PMC3873991; doi:10.1371/journal.pone.0084489)
Supplement: Table S6 — The association between the haplotypes in CCNB1 and Her2 status. (DOC) [file pone.0084489.s006.doc]

Table S6. The association between the haplotypes in CCNB1 and Her2 status.

| Gene | Haplotype | Her2 | | | | | |
| --- | --- | --- | --- | --- | --- | --- | --- |
|  |  | Negative | Positive | OR (95% CI) | P value | aOR (95% CI) | P value |
| CCNB1 | TGTT | 76.35% | 23.65% |  |  |  |  |
|  | CGGT | 70.58% | 29.42% | **1.346 (1.039-1.744)** | **0.025** | **1.340 (1.034-1.738)** | **0.027** |
|  | TAGT | 69.40% | 30.60% | **1.424 (1.059-1.391)** | **0.019** | **1.409 (1.048-1.895)** | **0.023** |
|  | TGTC | 74.81% | 25.19% | 1.087 (0.710-1.664) | 0.701 | 1.081 (0.706-1.657) | 0.720 |
|  | TGGT | 74.77% | 25.23% | 1.090 (0.681-1.743) | 0.720 | 1.088 (0.680-1.742) | 0.725 |
|  | else | 78.95% | 21.05% | 0.861 (0.387-1.915) | 0.714 | 0.861 (0.386-1.920) | 0.715 |
