# Supplementary material for: Association Study of Germline Variants in CCNB1 and CDK1 with Breast Cancer Susceptibility, Progression, and Survival among Chinese Han Women
Source: PLoS One. 2013 Dec 27;8(12):e84489. doi: 10.1371/journal.pone.0084489 (PMC3873991; doi:10.1371/journal.pone.0084489)
Supplement: Table S7 — The association between the diplotype in CCNB1 and Her2 status. (DOC) [file pone.0084489.s007.doc]

Table S7. The association between the diplotype in CCNB1 and Her2 status.

| Gene | Diplotype | Her2 | | | | | |
| --- | --- | --- | --- | --- | --- | --- | --- |
|  |  | Negative | Positive | OR (95% CI) | P value | aOR (95% CI) | P value |
| CCNB1 | TGTT/CGGT | 70.83% | 29.17% |  |  |  |  |
|  | TGTT/TGTT | 80.79% | 19.21% | **0.577 (0.402-0.828)** | **0.003** | **0.582 (0.405-0.836)** | **0.003** |
|  | TGTT/TAGT | 75.00% | 25.00% | 0.810 (0.554-1.182) | 0.274 | 0.803 (0.549-1.175) | 0.259 |
|  | CGGT/TAGT | 68.54% | 31.46% | 1.115 (0.758-1.639) | 0.581 | 1.113 (0.756-1.637) | 0.588 |
|  | CGGT/CGGT | 69.33% | 30.67% | 1.074 (0.712-1.620) | 0.733 | 1.078 (0.714-1.627) | 0.722 |
|  | TGTT/TGTC | 71.70% | 28.30% | 0.959 (0.595-1.544) | 0.862 | 0.961 (0.591-1.551) | 0.872 |
|  | CGGT/TGTC | 80.49% | 19.51% | 0.589 (0.327-1.061) | 0.078 | 0.582 (0.322-1.053) | 0.074 |
|  | TGTT/TGGT | 76.74% | 23.26% | 0.736 (0.426-1.271) | 0.272 | 0.736 (0.426-1.273) | 0.273 |
|  | CGGT/TGGT | 70.37% | 29.63% | 1.023 (0.548-1.909) | 0.944 | 1.002 (0.535-1.875) | 0.995 |
|  | TAGT/TAGT | 62.86% | 37.14% | 1.435 (0.843-2.444) | 0.184 | 1.407 (0.825-2.399) | 0.210 |
|  | TAGT/TGTC | 69.57% | 30.43% | 1.063 (0.546-2.067) | 0.858 | 1.049 (0.538-2.048) | 0.887 |
|  | TAGT/TGGT | 66.67% | 33.33% | 1.214 (0.587-2.512) | 0.601 | 1.225 (0.590-2.543) | 0.586 |
|  | else | 78.26% | 21.74% | 0.675 (0.392-1.160) | 0.155 | 0.676 (0.392-1.165) | 0.159 |
