# Supplementary material for: Association Study of Germline Variants in CCNB1 and CDK1 with Breast Cancer Susceptibility, Progression, and Survival among Chinese Han Women
Source: PLoS One. 2013 Dec 27;8(12):e84489. doi: 10.1371/journal.pone.0084489 (PMC3873991; doi:10.1371/journal.pone.0084489)
Supplement: Table S8 — The association between the haplotypes in CDK1 and PR status. (DOC) [file pone.0084489.s008.doc]

Table S8. The association between the haplotypes in CDK1 and PR status.

| Gene | SNP | PR | | | | | | | |
| --- | --- | --- | --- | --- | --- | --- | --- | --- | --- |
|  |  | χ^2^ | | | Ptrend | Logistical Regression | | | |
|  |  | positive | negative | P value |  | OR (95% CI) | P value | aOR (95% CI) | P value |
| CDK1 | rs2448343(G>A) |  |  |  |  |  |  |  |  |
|  | GG | 377 (63.26%) | 214 (70.39%) | 0.094 | **0.008** |  |  |  |  |
|  | AG | 196 (32.89%) | 79 (25.99%) |  |  | **0.710 (0.521-0.969)** | **0.031** | **0.682 (0.498-0.934)** | **0.017** |
|  | AA | 23 (3.86%) | 11 (2.62%) |  |  | 0.843 (0.403-1.762) | 0.649 | 0.823 (0.390-1.739) | 0.610 |
|  | rs3213048(T>C) |  |  |  |  |  |  |  |  |
|  | TT | 251 (42.11%) | 104 (34.21%) | 0.045 | **0.001** |  |  |  |  |
|  | CT | 266 (44.63%) | 147 (48.36%) |  |  | 1.334 (0.983-1.809) | 0.064 | 1.346 (0.987-1.836) | 0.061 |
|  | CC | 79 (13.26%) | 53 (17.43%) |  |  | **1.619 (1.068-2.455)** | **0.023** | **1.690 (1.101-2.592)** | **0.016** |
|  | rs3213067(A>G) |  |  |  |  |  |  |  |  |
|  | AA | 438 (73.49%) | 204 (67.11%) | 0.096 | **0.021** |  |  |  |  |
|  | GA | 137 (22.99%) | 90 (29.61%) |  |  | **1.410 (1.031-1.930)** | **0.032** | **1.431 (1.040-1.968)** | **0.028** |
|  | GG | 21 (73.49%) | 10 (3.29%) |  |  | 1.022 (0.473-2.211) | 0.955 | 1.074 (0.491-2.348) | 0.857 |
|  | rs1871446(C>T) |  |  |  |  |  |  |  |  |
|  | CC | 456 (76.51%) | 244 (80.26%) | 0.427 | 0.330 |  |  |  |  |
|  | TC | 132 (22.15%) | 56 (18.42%) |  |  | 0.793 (0.559-1.124) | 0.193 | 0.782 (0.549-1.113) | 0.172 |
|  | TT | 8 (1.34%) | 4 (1.32%) |  |  | 0.934 (0.279-3.134) | 0.913 | 0.919 (0.267-3.161) | 0.893 |
|  | rs10711(G>T) |  |  |  |  |  |  |  |  |
|  | GG | 259 (43.46%) | 129 (42.43%) | 0.173 | 0.145 |  |  |  |  |
|  | TG | 234 (39.26%) | 135 (44.41%) |  |  | 1.158 (0.859-1.562) | 0.336 | 1.105 (0.816-1.496) | 0.518 |
|  | TT | 103 (17.28%) | 40 (13.16%) |  |  | 0.780 (0.511-1.189) | 0.248 | 0.802 (0.524-1.229) | 0.312 |
|  | rs1060343(G>A) |  |  |  |  |  |  |  |  |
|  | GG | 466 (78.19%) | 248 (81.58%) | 0.483 | 0.375 |  |  |  |  |
|  | AG | 124 (20.81%) | 53 (17.43%) |  |  | 0.803 (0.562-1.147) | 0.228 | 0.788 (0.549-1.130) | 0.195 |
|  | AA | 6 (1.01%) | 3 (0.99%) |  |  | 0.940 (0.233-3.789) | 0.930 | 0.876 (0.214-3.580) | 0.854 |
