# Supplementary material for: Association Study of Germline Variants in CCNB1 and CDK1 with Breast Cancer Susceptibility, Progression, and Survival among Chinese Han Women
Source: PLoS One. 2013 Dec 27;8(12):e84489. doi: 10.1371/journal.pone.0084489 (PMC3873991; doi:10.1371/journal.pone.0084489)
Supplement: Table S9 — The association between the haplotypes in CDK1 and PR status. (DOC) [file pone.0084489.s009.doc]

Table S9. The association between the haplotypes in CDK1 and PR status.

| Gene | Haplotype | PR | | | | | |
| --- | --- | --- | --- | --- | --- | --- | --- |
|  |  | Positive | Negative | OR (95% CI) | P value | aOR (95% CI) | P value |
| CDK1 | GCACG | 62.54% | 37.46% |  |  |  |  |
|  | GTACG | 69.81% | 30.19% | **0.721 (0.558-0.931)** | **0.012** | **0.697 (0.537-0.905)** | **0.007** |
|  | GTGCT | 63.35% | 36.65% | 0.964 (0.711-1.307) | 0.815 | 0.951 (0.698-1.296) | 0.751 |
|  | ATATT | 67.44% | 32.56% | 0.805 (0.562-1.151) | 0.234 | 0.790 (0.548-1.137) | 0.205 |
|  | ATACT | 76.69% | 23.31% | **0.507 (0.328-0.782)** | **0.002** | **0.481 (0.310-0.748)** | **0.001** |
|  | GTATT | 90.91% | 9.09% | **0.167 (0.039-0.720)** | **0.016** | **0.174 (0.040-0.760)** | **0.020** |
|  | else | 60.43% | 39.57% | 1.091 (0.748-1.591) | 0.650 | 1.073 (0.731-1.575) | 0.718 |
